# Conditional Random Field-Guided Multi-Focus Image Fusion

**DOI:** 10.3390/jimaging8090240

**Published:** 2022-09-05

**Authors:** Odysseas Bouzos, Ioannis Andreadis, Nikolaos Mitianoudis

**Affiliations:** Department of Electrical and Computer Engineering, Democritus University of Thrace, 67100 Xanthi, Greece

**Keywords:** multi-focus, image fusion, transform domain, graphical model

## Abstract

Multi-Focus image fusion is of great importance in order to cope with the limited Depth-of-Field of optical lenses. Since input images contain noise, multi-focus image fusion methods that support denoising are important. Transform-domain methods have been applied to image fusion, however, they are likely to produce artifacts. In order to cope with these issues, we introduce the Conditional Random Field (CRF) CRF-Guided fusion method. A novel Edge Aware Centering method is proposed and employed to extract the low and high frequencies of the input images. The Independent Component Analysis—ICA transform is applied to high-frequency components and a Conditional Random Field (CRF) model is created from the low frequency and the transform coefficients. The CRF model is solved efficiently with the α-expansion method. The estimated labels are used to guide the fusion of the low-frequency components and the transform coefficients. Inverse ICA is then applied to the fused transform coefficients. Finally, the fused image is the addition of the fused low frequency and the fused high frequency. CRF-Guided fusion does not introduce artifacts during fusion and supports image denoising during fusion by applying transform domain coefficient shrinkage. Quantitative and qualitative evaluation demonstrate the superior performance of CRF-Guided fusion compared to state-of-the-art multi-focus image fusion methods.

## 1. Introduction

The limited Depth-of-Field of optical lenses allows only parts of the scene within a certain distance from the camera sensor to be captured well-focused each time, while the remaining parts of the scene stay out-of-focus or blurred. Multi-focus image fusion algorithms are thus of vital importance in order to cope with this limitation. Multi-focus image fusion methods merge multiple input images captured with different focus settings into a single image with extended Depth-of-Field. More precisely, the well-focused pixels of the input images are preserved in the fused image and the out-of-focus pixels of the input images are discarded. Consequently, the fused image should have extended Depth-of-Field and thus more information than each one of the input images and should not introduce artifacts during fusion.

The problem of multi-focus image fusion has been explored widely in the literature. Lately, a number of multi-focus image fusion methods have been proposed. Liu et al. [1] classified the multi-focus image fusion methods in four categories: spatial-domain methods, transform-domain methods, combined methods and deep learning methods. In spatial-domain methods, the fused image is estimated as the weighted average of the input images. Spatial-domain methods are also classified as block-based, region-based, and pixel-based. In block-based methods, the image is decomposed into blocks of fixed size, and the activity level is estimated individually for each of these blocks.

However, since blocks are likely to contain both well-focused and out-of-focus pixels, the block-based methods are likely to have blocking artifacts near the boundaries of well-focused and out-of-focus pixels. Thus, the fused image has lower quality near their boundary. Region-based methods, use a whole region of irregular shape in order to estimate the saliency of the included pixels. Although region-based methods provide higher flexibility than block-based methods, a region may also contain simultaneously both well-focused and out-of-focus pixels. As a result, region-based methods also produce artifacts and have lower fused image quality near the boundaries of well-focused and out-of-focus pixels. In order to overcome these issues, pixel-based methods have lately gained more popularity. In these methods, activity level estimation is carried out at pixel level. Pixel-based methods do not have blocking artifacts and have better accuracy near the boundary of well-focused and out-of-focus pixels, however, they are likely to produce noisy weight maps, which also lead to fused images of lower image quality. Popular spatial domain-based multi-focus image fusion methods include: Quadtree [2], Boundary Finding [3], dense Sift [4], guided filtering [5], PCNN [6] and Image Matting [7]. Singh et al. [8] used the Arithmetic optimization algorithm (AOA) in order to estimate the weight maps for image fusion, which were refined with weighted least square optimization (WLS). The fused image is extracted through pixel-wise weighted average fusion. In [9], the fusion method FNMRA was presented, which used the modified naked mole-rat algorithm (mNMRA) in order to generate the weight maps, which were refined with weighted least-squares optimization. Pixel-wise single-scale composition was used in order to create the fused image.

In transform-domain methods, a forward transform is firstly applied to the input images. A fusion rule is then applied in order to combine the transform coefficients. Finally, an inverse transform is applied to the fused coefficients in order to return the fused image to the spatial domain. An advantage of dictionary-based transform-domain methods is the support of image denoising during fusion, by applying shrinkage methods, such as [10], which can be used to remove the noisy transform-domain coefficients. An issue of transform-domain methods lies in the imperfect forward-backward transforms that result in visible artifacts due to the Gibbs phenomenon. Since both the selection of the transform domain and the manual design of the fusion rule highly impact the quality of the fused image a number of transform domain-based multi-focus image fusion methods have been introduced. Typical transform domain-based multi-focus image fusion methods include: ICA [11], ASR [12], CSR [13], NSCT [14], NSCT-SR [15], MWGF [16] and DCHWT [17]. Qin et al. [18] proposed a new image fusion method combining discrete wavelet transform (DWT) and sparse representation (SR). Jagtap et al. [19] introduced information preservation-based guided filtering in order to decompose the input images into base and detail images. Low-rank representation was used in order to estimate the focus map and perform a fusion of the detailed images. In [20], the authors used weight maps based on local contrast, and the fused image was estimated with multi-scale weighted average fusion based on pyramid decomposition.

The methods that lie in the combined methods category employ both the merits of spatial and transform domain methods. Nonetheless, each method uses different domains. Bouzos et al. [21] combined the advantages of both the ICA domain and spatial domain. Chai et al. [22] combined advantages of multi-scale decomposition and spatial domain. He et al. [23] combined the Meanshift algorithm and NSCT domain. An issue of the aforementioned methods is that they do not support image denoising during fusion. Singh et al. [24] proposed the Discrete Wavelet Transform-bilateral filter (DWTBF) method, which combined the Discrete Wavelet Transform (DWT) and the bilateral filter. In [25], the authors combined a multi-resolution pyramid and the bilateral filter in order to predict the fused image.

Lately, deep learning-based methods have gained more popularity. According to the study [26], deep learning-based methods, are classified into decision map-based methods and end-to-end methods. In decision-map-based methods, the deep learning networks predict a decision map, with a classification-based architecture. Post-processing steps, including morphological operations, are usually employed to refine the decision map. The decision map is later used to guide the fusion of the input images, by selecting the respective pixels from the input images. Typical decision map-based deep learning multi-focus image fusion methods include: CNNFusion [27], ECNN [28] and p-CNN [29]. On the other hand, end-to-end deep learning-based networks, directly predict the fused image without the intermediate step of the decision map. Typical end-to-end based deep learning networks for multi-focus image fusion include: IFCNN [30] and DenseFuse [31]. Ma et al. [32] introduced a multi-focus image fusion method based on an end-to-end multi-scale generative adversarial network (MsGAN). Wei et al. [33] combined advantages of sparse representation and CNN networks in order to estimate the fusion weights for the multi-focus image fusion problem. Since the sensitivity of the aforementioned deep learning-based methods to noise was not studied, the methods are likely to be sensitive to noise. In addition, these deep learning-based multi-focus image fusion methods do not support image denoising during fusion.

In this manuscript, we introduce CRF-Guided fusion, which is a novel transform domain-based method that uses the Conditional Random Field model, in order to guide the fusion of the transform-domain ICA method. Due to various sources, input images are likely to contain noise, thus multi-focus methods that are robust to noise and support fusion and denoising during fusion are of great importance. Since CRF-Guided fusion is a dictionary-based method (ICA), the method is robust to Gaussian noise and supports image denoising during fusion by applying the shrinkage coefficient method [10]. A novel Edge Aware Centering method (EAC) is also introduced and is used, instead of the typical centering method, and alleviates artifacts caused by the centering procedure. The combination of EAC and the proposed CRF-Guided fusion method produce fused images of high quality, without introducing artifacts for both clean images and images that contain Gaussian noise, while also supporting denoising during fusion.

The main contributions of this manuscript and improvements over our previous method [21] are:

1. the development of the novel EAC method, which preserves the strong edges of the input images, instead of the typical centering method.
2. the design of a novel framework, based on a CRF model, that is suitable for transform-domain image fusion.
3. the design of a novel transform-domain fusion method that produces fused images of high visual quality, preserves via CRF optimization, the boundary between well-focused and out-of-focus pixels, and does not introduce artifacts during fusion.
4. the introduction of a novel transform-domain fusion rule, based on the labels extracted from the CRF model, that produces fused images of higher image quality without the transform-domain artifacts.
5. the robustness of the proposed method against Gaussian noise and the support of denoising during fusion, by applying the transform-domain coefficient shrinkage method [10].

## 2. Proposed Method Description

**Figure 1:**
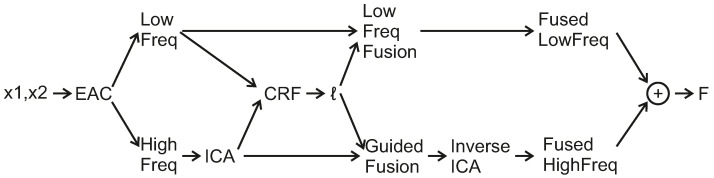
CRF-Guided fusion Framework for input images $x_{1},x_{2}$, labels *ℓ* as estimated from the CRF minimization, and the fused image *F* is constructed by the addition of the low-frequency and high-frequency fusion results.

**Figure 2:**
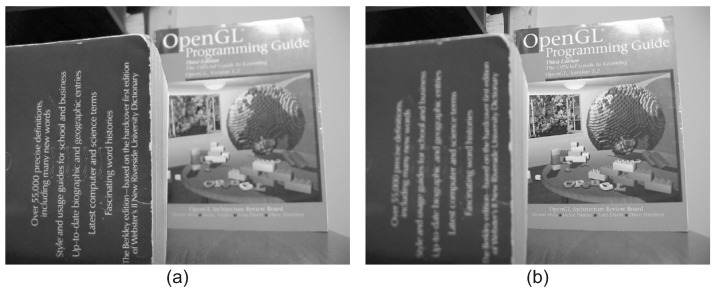
Source input images: (**a**) Near focused image, (**b**) Far focused image.

The proposed framework of the CRF-Guided fusion is summarised in Figure 1. An outline of the method is now provided: Firstly, Edge Aware Centering is applied to the input images, in order to extract the low and high-frequency components. The Forward ICA transform is then applied to the high frequencies of the input images. Then, the Low frequency and ICA coefficients are used to compute the Unary *U* and Smoothness *V* potentials and thus construct the CRF model. Consequently, the CRF model is solved efficiently with the α-expansion method based on GraphCuts [34]. The predicted labels are then employed to fuse the low frequencies leading to the fused low-frequency image. In addition, they are also used to guide the fusion of the transform-domain ICA coefficients. Lastly, the inverse ICA transform is applied to the fused transform coefficients in order to return the fused high-frequency component. Finally, the fused image *F* is estimated by the addition of the fused low-frequency and the fused high-frequency components. More details of the aforementioned steps of the proposed framework are included in the following subsections. Figure 2 includes two source input images for multi-focus image fusion that will be used during the steps of the CRF-Guided fusion.

### 2.1. Edge Aware Centering

In this section, we introduce the *Edge Aware Centering* (EAC) method, which is used instead of the typical centering method, in order to estimate the low frequency of the multi-focus input images. EAC consists of a spatially varying Gaussian filter that preserves the strong edges of the input images. More precisely,

(1) $$w_{i,j}=\exp\left(-\frac{{\left(x_{i,j}-\mu_{i,j}\right)}^{2}}{2\langle {\left(x_{m,n}-\mu_{i,j}\right)}^{2}\rangle }\right)$$

where $w_{i,j}$ is the weight at spatial location $\left(i,j\right)$, $\mu_{i,j}$ is the mean value of a 7×7 block with central pixel at $\left(i,j\right)$, *x* is the input image and $m\in \left[i-3,i+3\right],n\in \left[j-3,j+3\right]$. In addition, the $\langle \cdot \rangle$ operator implies averaging over the all m,n values. Finally, the filtered image *f* in spatial locations $\left(i,j\right)$ is estimated as:

(2) $$f_{i,j}=\frac{\sum_{m,n}w_{m,n}I_{mn}}{\sum_{m,n}w_{m,n}}$$

**Figure 3:**
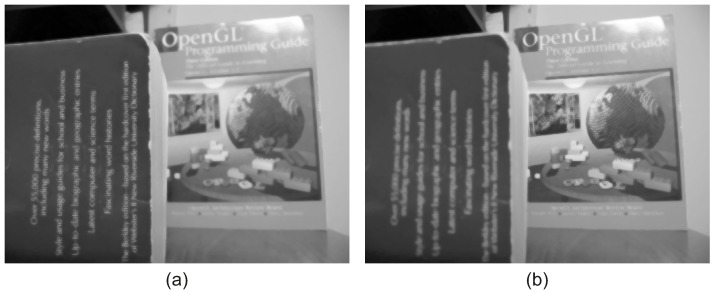
Low frequency of input images using the EAC: (**a**) Low frequency of near focused image, (**b**) Low frequency of far focused image. It is evident that the EAC preserves the strong image edges.

EAC is applied to both input images in order to estimate the low frequency of each image. Figure 3 includes the low-frequency images, as computed by applying the proposed EAC to the input images of Figure 2. It is evident that the EAC preserves accurately the strong edges of the input images.

**Figure 4:**
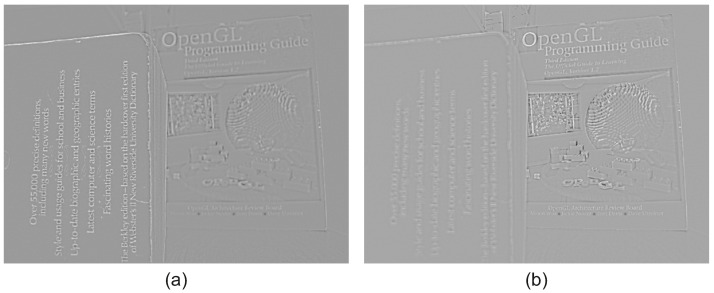
High frequency of input images: (**a**) High frequency of near focused image, (**b**) High frequency of far focused image.

By subtracting the low-frequency images from the input images, we extract the high-frequency images as demonstrated in Figure 4. The forward ICA transform is then applied to the high-frequency images in order to get the transform domain coefficients. For more information on the estimation of the ICA transform, and its application on images for fusion, please refer to [11].

### 2.2. Energy Minimization

In order to model the multi-focus image fusion problem and solve it efficiently, we construct an energy minimization equation. Since solvers of graph cuts can reach a global or close-to-global optimum solution, we formulate the energy minimization problem of multi-focus image fusion as a graph cut problem. More precisely, we introduce the Conditional Random Field (CRF) equation that describes our multi-focus image fusion problem, which is solved efficiently with the inference method of α-expansion reaching a global or close-to-global optimum solution. The solution of the proposed energy minimization leads to the optimum labels of the decision that is used to guide the fusion of low frequency and transform coefficients.

In order to guide the fusion of the low frequency and the transform coefficients, we formulate the Conditional Random Field (CRF) equation, as follows:

(3) $$\ell =\arg\min_{\ell }\left[\sum_{i=1}^{N}U\left(\ell_{i}\right)+\sum_{\left(m,n\right)\in C}V_{m,n}\left(\ell_{m},\ell_{n}\right)\right]$$

where *ℓ* are the estimated labels, *U* is the unary potential function, *V* is the pairwise potential function, *i* are spatial locations, and m,n adjacent pixels in the *C* which is the N8-neighborhood. The energy minimization equation is optimized using the α-expansion method, based on GraphCuts [34].

### 2.3. Inference α-Expansion Method

In the α-expansion, the optimization problem is divided into a sequence of binary-valued maximization problems. Given a current label configuration *h* and a fixed label α∈U, with *U* being the set of all label values. In the α-expansion move, each pixel *i* gets a binary decision, to either retain its old value or change it to label α. The expansion move starts with the initial set of labels $h^{0}$ and then based on some order, computes the optimal α- expansion moves for the labels α. Only the moves that lead to the increase of the objective function are accepted.

### 2.4. Unary Potential Estimation

**Figure 5:**
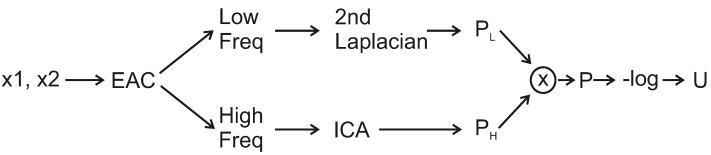
Unary potential estimation for CRF-Guided method.

Let us assume that $x_{1},x_{2}$ are the input images, $P_{L}$ is the probability of low frequency, $P_{H}$ the probability of high frequency, *P* the probability of the input images, and *U* unary potential function. Figure 5 depicts the method of estimating the unary potential. More precisely, EAC is firstly applied to the images to extract low and high frequencies. The 2nd Laplacian is applied to both low-frequency components and the probability of the low frequency, $P_{L}$ is estimated by:

(4) $$P_{L}\left(\ell_{n}\right)=\left\{\begin{array}{cc} \; & \frac{S_{0}}{S_{0}+S_{1}},\;\ell_{n}=0\\ \; & \frac{S_{1}}{S_{0}+S_{1}},\;\ell_{n}=1 \end{array}\right.$$

where, $S_{0},S_{1}$ are the second Laplacian of the low frequencies of the first and the second image respectively.

The probability of the high frequency $P_{H}$ is extracted by the ICA coefficients and is estimated as follows:

(5) $$P_{H}\left(\ell_{n}\right)=\left\{\begin{array}{cc} \; & \frac{∥C_{0}∥}{∥C_{0}∥+∥C_{1}∥},\ell_{n}=0\\ \; & \frac{∥C_{1}∥}{∥C_{0}∥+∥C_{1}∥},\ell_{n}=1 \end{array}\right.$$

where $∥C_{0}∥$ is the L2-norm of ICA coefficients of the first image, $∥C_{1}∥$ is the L2-norm of ICA coefficients of the second image. In order to determine the probability that each one of the input images *i* should contribute to the spatial location *n* of the guidance map, we compute the combined probability of high and low frequencies for each image. This probability we call the probability of input image that corresponds to label *ℓ*. Thus probability of each input image $P\left(\ell_{n}\right)$ is estimated as follows:

(6) $$P\left(\ell_{n}\right)=P_{H}\left(\ell_{n}\right)*P_{L}\left(\ell_{n}\right)$$

Finally, the Unary potential function *U* is estimated by the negative likelihood of the predicted probabilities:

(7) $$U\left(\ell_{n}\right)=-\log P\left(\ell_{n}\right)$$

### 2.5. Smoothness Term

The smoothness potential function *V* is estimated from the low-frequency image, as follows:

(8) $$V_{pq}=\frac{\left|l_{0}^{p}-l_{1}^{q}\right|+\left|l_{1}^{p}-l_{0}^{q}\right|}{\left|l_{0}^{p}-l_{0}^{q}\right|+\left|l_{1}^{p}-l_{1}^{q}\right|}$$

where p,q are adjacent pixels in the N8-neighborhood and $l_{0},l_{1}$ are the first and second low-frequency images respectively. Finally, the labels *ℓ* of the CRF model in (3) are estimated efficiently using the α-expansion method [34].

**Figure 6:**
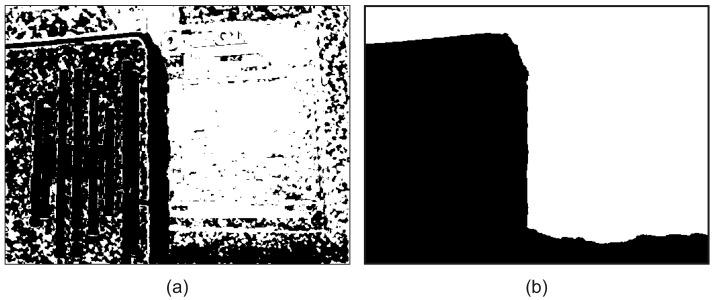
Predicted labels, black pixels correspond to ℓ=0, white pixels correspond to ℓ=1, (**a**) ℓ=argminU, (**b**) $\ell =\arg\min\left(CRF\right)$.

Figure 6 demonstrates the labels, as estimated from the direct minimization of the unary term *U* and the labels, as estimated from the CRF minimization (3). The predicted labels are then used to fuse the low frequency of the input images.

(9) $$L_{F}\left(i\right)=\left(1-\ell_{i}\right)*L_{0}\left(i\right)+\ell_{i}*L_{1}\left(i\right)$$

where $L_{F}$ is the low-frequency fused image, *i* is the spatial location, $L_{0}$ is the low frequency of the first image and $L_{1}$ is the low- frequency of the second image.

### 2.6. Transform-Domain CRF Fusion Rule

**Figure 7:**
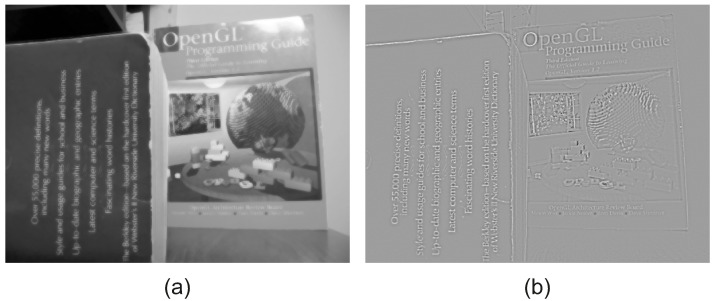
(**a**) Fused low frequency, (**b**) Fused high frequency.

A sliding window with size 7×7 is applied to the decision map of the predicted probabilities. The transform coefficients that correspond to each 7×7 block are then fused according to the label of the central pixel of the block by selecting the respective coefficients from the input images that correspond to that label. Inverse ICA is then applied to the fused transform coefficients in order to return the fused high frequency. Figure 7 depicts the fused low-frequency component and the fused high-frequency component.

**Figure 8:**
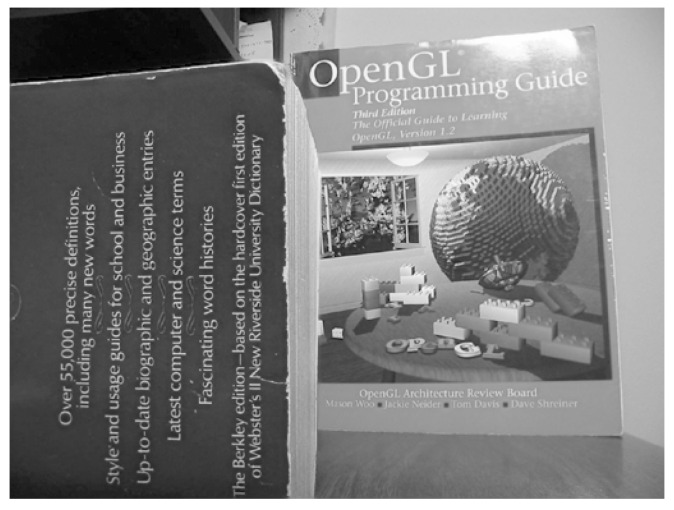
Final fused image by the proposed method.

Finally, the fused image is estimated by the addition of the low and high-frequency components. Figure 8 demonstrates the final fused image.

## 3. Fusion and Denoising

A major advantage of the proposed CRF-Guided fusion is the robustness against Gaussian noise and the support of denoising during fusion. In the case of Gaussian noise, the coefficient shrinkage method [10] is applied to the transform coefficients of both input images. More precisely,

(10) $$C\left(k\right)=0,\;\mathrm{if}\left|C(k)\right|<1.95*\sigma_{n}$$

where C(k) is the *k*-th transform coefficient in the ICA domain and $\sigma_{n}$ is the standard deviation of the noise, which is estimated by areas of the image where there is low activity. Low activity areas contain no strong edges, therefore may contain only noise and thus can be used to estimate the noise standard deviation $\sigma_{n}$. The denoised transform coefficients are then employed to estimate the $P_{H}$ of both input images. Consequently, Guided fusion from the CRF labels is performed on the denoised transform coefficients. Then, the inverse ICA transform is used to return the denoised high-frequency image. Lastly, the final denoised fused image is formed by the addition of the denoised high-frequency and the fused low-frequency images.

**Figure 9:**
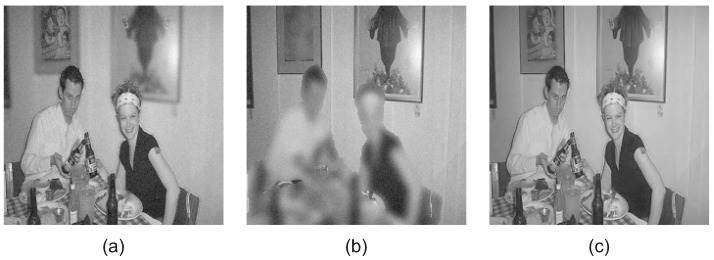
(**a**) Near-focused image with Gaussian noise $\sigma_{n}=5$, (**b**) Far-focused image with Gaussian noise $\sigma_{n}=5$, (**c**) Denoised fused image.

Figure 9 includes the noisy input images with Gaussian noise $N\left(0,\sigma^{2}\right)$, σ=5 and the denoised fused image *F*. The fused image *F* is successfully denoised during the fusion, as is demonstrated in Figure 9c.

**Figure 10:**
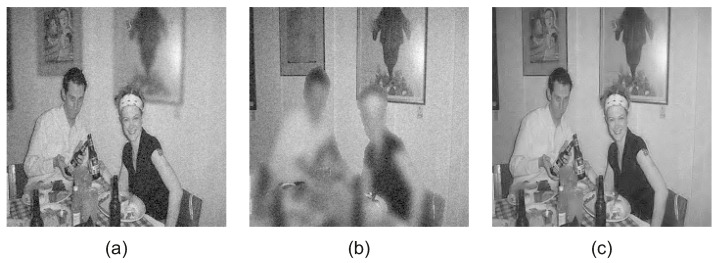
(**a**) Near focused image with Gaussian noise σ=10, (**b**) Far focused image with Gaussian noise σ=10, (**c**) Denoised fused image.

Figure 10 includes the noisy input images with Gaussian noise $N\left(0,\sigma^{2}\right)$, σ=10 and the denoised fused image *F*. The proposed CRF-Guided fusion framework can successfully produce the denoised fused image Figure 10c, with denoising performed during fusion.

## 4. Experimental Results

The proposed CRF-Guided fusion method is compared to 13 state-of-the-art image fusion methods in the two public datasets: the Lytro dataset [35], which consists of 20 color input image pairs and the grayscale dataset [3], which consists of 17 grayscale input image pairs. The state-of-the-art compared methods are: GBM [36], NSCT [14], ICA [11], DCHWT [17], ASR [12], IFCNN [30] and DenseFuse [31], acof [37], CFL [38], ConvCFL [39], DTNP [40], MLCF [41] and Joint [42]. Both quantitative and qualitative results are included in order to evaluate the performance of CRF-Guided fusion and the compared multi-focus image fusion methods.

### 4.1. Quantitative Evaluation

In [43,44] Singh et al. made a review of multiple image fusion algorithms along with the image fusion performance metrics. In order to assess the quality of the fused images of the compared multi-focus image fusion methods, eight metrics are used. More precisely the metrics used are: Mutual Information (MI) [45], $Q_{ab/f}$ [46], Qg [47], Qy [48], CB [49], SSIM [50], NIQE [51] and Entropy.

#### 4.1.1. Mutual Information—MI

Mutual Information—MI is an information theory-based metric and the objective measure of the mutual dependence of two random variables. For two discrete random variables *U* and *V*, MI is defined as follows:

(11) $$MI\left(U;V\right)=\sum_{v\in V}\sum_{u\in U}p\left(u,v\right)\log_{2}\frac{p\left(u,v\right)}{p\left(u\right)p\left(v\right)}$$

#### 4.1.2. Yang’s Metric Qy

Yang et al. [48] proposed the image structural similarity-based metric $Q_{Y}$. For input images A,B and fused image *F*, it is defined as follows:

(12) $$Q_{Y}=\left\{\begin{array}{cc} \lambda \left(w\right)SSIM\left(A,F|w\right)+\left(1-\lambda \left(w\right)\right)SSIM\left(B,F|w\right), & SSIM(A,B|w)⩾0.75\\ \max\left\{SSIM\left(A,F|w\right),SSIM\left(B,F|w\right)\right\}, & SSIM\left(A,B|w\right)<0.75 \end{array}\right.$$

(13) $$\lambda \left(w\right)=\frac{s\left(A|w\right)}{s\left(A|w\right)+s\left(B|w\right)}$$

where $s\left(A|w\right)$ is a local salience measure of image *A* within a window *w*. A higher value of $Q_{Y}$ indicates better-fused image quality and higher structural similarity of the fused images and the input images.

#### 4.1.3. Chen-Blum Metric—CB

The Chen-Blum Metric CB [49] is a human perception-inspired fusion metric that features the following five steps:

1. Contrast sensitivity filtering: Filtered image $I_{A}^{\prime }\left(m,n\right)=I_{A}\left(m,n\right)S\left(r\right)$, where $S\left(r\right)$ is the CSF filter in polar form and $r=\sqrt{m^{2}+n^{2}}$.
2. Local contrast computation:
  (14) $$C\left(i,j\right)=\frac{\phi_{k}\left(i,j\right)*I\left(i,j\right)}{\phi_{k+1}\left(i,j\right)*I\left(i,j\right)}-1$$
  (15) $$\phi_{k}\left(x,y\right)=\frac{1}{{\left(\sqrt{2\pi }\sigma_{k}\right)}^{2}}e^{-\frac{x^{2}+y^{2}}{2\sigma_{k}^{2}}}$$
  where $\sigma_{k}=2$.
3. Contrast preservation calculation: For input image $I_{A}$ the masked contrast map is estimated as:
  (16) $$C_{A}^{\prime }=\frac{t{\left(C_{A}\right)}^{p}}{h{\left(C_{A}\right)}^{q}+Z}$$
  where t,h,p,q,Z are real scalar parameters that determine the shape of the nonlinearity of the masking function [49].
4. Generation of saliency map: The saliency map for image $I_{A}$ is:
  (17) $$\lambda_{A}=\frac{{C_{A}^{\prime }}^{2}}{{C_{A}^{\prime }}^{2}+{C_{B}^{\prime }}^{2}}$$
  The value of information preservation is:
  (18) $$Q_{AF}=\left\{\begin{array}{cc} \frac{C_{A}^{\prime }}{C_{F}^{\prime }} & \mathrm{if}C_{A}^{\prime }<C_{F}^{\prime }\\ \frac{C_{F}^{\prime }}{C_{A}^{\prime }} & \mathrm{otherwise}. \end{array}\right.$$
5. The global quality map is defined as:
  (19) $$Q_{GQM}\left(i,j\right)=\lambda_{A}\left(i,j\right)Q_{AF}\left(i,j\right)+\lambda_{B}\left(i,j\right)Q_{BF}\left(i,j\right)$$
  The value of metric CB is the average of the global quality map:
  (20) $$CB={\mathrm{mean}}_{i,j}Q_{GQM}\left(i,j\right)$$

#### 4.1.4. Gradient Based Methods—$Q_{G}$, $Q_{AB/F}$

Xydeas et al. [47] proposed a metric to measure the amount of edge information from source images to the fused image. $Q_{G}$ is a gradient-based method. Firstly, a Sobel operator is applied to input image *A* to extract edge strength $g_{A}\left(i,j\right)$ and orientation $\alpha_{A}\left(i,j\right)$.

(21) $$g_{a}\left(i,j\right)=\sqrt{s_{A}^{x}{\left(i,j\right)}^{2}+s_{A}^{y}{\left(i,j\right)}^{2}}.$$

(22) $$\alpha_{A}\left(i,j\right)=\tan^{-1}\left(\frac{s_{A}^{y}\left(i,j\right)}{s_{A}^{x}\left(i,j\right)}\right)$$

where $s_{A}^{x},s_{A}^{y}$ are the outputs of the convolution application of the horizontal and vertical Sobel templates respectively. The relative strength between input image *A* and fused image *F* is:

(23) $$G^{AF}\left(i,j\right)=\left\{\begin{array}{cc} \frac{g_{F}\left(i,j\right)}{g_{A}\left(i,j\right)}, & \mathrm{if}g_{A}\left(i,j\right)>g_{F}\left(i,j\right)\\ \frac{g_{A}\left(i,j\right)}{g_{F}\left(i,j\right)}, & \mathrm{otherwise}. \end{array}\right.$$

The orientation values $\Delta^{AF}$ between input image *A* and fused image *F* are:

(24) $$\Delta^{AF}\left(i,j\right)=1-\frac{\left|\alpha_{A}\left(i,j\right)-\alpha_{F}\left(i,j\right)\right|}{\pi /2}$$

The edge strength value is estimated as:

(25) $$Q_{g}^{AF}\left(i,j\right)=\frac{\Gamma_{g}}{1+e^{k_{g}\left(G^{AF}\left(i,j\right)-\sigma_{g}\right)}}$$

The orientation preservation value is estimated as:

(26) $$Q_{\alpha }^{AF}\left(i,j\right)=\frac{\Gamma_{\alpha }}{1+e^{k_{a}\left(\Delta^{AF}\left(i,j\right)-\sigma_{\alpha }\right)}}$$

The constants $\Gamma_{g},k_{g},\sigma_{g}$ and $\Gamma_{\alpha },k_{\alpha },\sigma_{\alpha }$ are used to define the shape of the sigmoid functions used for the edge strength and orientation preservation values [47].

(27) $$Q^{AB/F}=\frac{\sum_{n=1}^{N}\sum_{m=1}^{M}\left[Q^{AF}w^{A}+Q^{AB}w^{B}\right]}{\sum_{n=1}^{N}\sum_{m=1}^{M}\left[w^{A}+w^{B}\right]}$$

and

(28) $$Q^{AF}=Q_{g}^{AF}Q_{\alpha }^{AF}$$

where $Q^{AF}\left(i,j\right)$ denotes the edge similarity at position $\left(i,j\right)$ between input image *A* and fused image *F*, $Q_{g}^{AF}$ the edge strength similarity and $Q_{\alpha }^{AF}$ the orientation similarity.

#### 4.1.5. Structural Similarity Index—SSIM [50]

The structural similarity index—SSIM for two images A,B is defined as:

(29) $$SSIM\left(A,B\right)=\frac{\left(2\mu_{A}\mu_{B}+C_{1}\right)\left(2*\sigma_{AB}+C_{2}\right)}{\left(\mu_{A}^{2}+\mu_{B}^{2}+C_{1}\right)\left(\sigma_{A}^{2}+\sigma_{B}^{2}+C_{2}\right)}$$

where $\mu_{A},\mu_{B}$ are the mean intensity values of images A,B, $\sigma_{A},\sigma_{B}$ are the standard deviation of images A,B and $\sigma_{AB}$ is the square root of covariance of A,B. $C_{1},C_{2}$ are constants. Due to the lack of ground truth image, the SSIM for input images A,B and fused image *F* in the experiments is defined as follows:

(30) $$SSIM=\frac{SSIM\left(A,F\right)+SSIM\left(B,F\right)}{2}$$

where A and B are the two input images and *F* is the fused image.

#### 4.1.6. Niqe [51]

NIQE is a blind image quality metric based on the Multivariate Gaussian Model (MVG). The quality of the fused image is defined as the distance between the quality aware natural scene statistic (NSS) model and the MVG fit, extracted from features of the distorted image:

(31) $$D\left(v_{1},v_{2},\Sigma_{1},\Sigma_{2}\right)=\sqrt{\left(\left({\left(v_{1}-v_{2}\right)}^{T}\right){\left(\frac{\Sigma_{1}+\Sigma_{2}}{2}\right)}^{-1}\left(v_{1}-v_{2}\right)\right)}$$

where $v_{1},v_{2}$ and $\Sigma_{1},\Sigma_{2}$ are the mean vectors and covariance matrices of the natural multivariate Gaussian model [51] and the multivariate Gaussian model that is fit to the fused image.

#### 4.1.7. Entropy

The entropy of an image *I* is defined as:

(32) $$E_{I}=-\sum_{j=1}^{2^{L}-1}p\left(s_{j}\right)\log_{2}\left(p\left(s_{j}\right)\right)$$

where *L* is the number of gray levels, $p\left(s_{j}\right)$ is the probability of occurrence of gray level $s_{j}$ in image *I*.

Table 1 includes the objective evaluation of the compared methods for the Lytro dataset [35].

For the Lytro dataset [35], the proposed CRF-Guided fusion method has the highest value for the metrics MI, $Q_{g}$, $Q^{AB/F}$, $Q_{Y}$, CB, the lowest value for the NIQE metric and the second highest score for SSIM and entropy. These results indicate that the fused quality of the proposed fused image is better than the compared state-of-the art methods. Since CRF-Guided has the highest Mutual Information [45], the proposed method preserves best the information of the input images. In addition, CRF-Guided has the highest Qg [47] and $Q^{AB/F}$ [46] values, which indicate that the proposed method preserves best the edge information from the input images to the fused image. In order to assess the quality of the structural similarity of the fused images, Yang’s metric $Q_{Y}$ [48] and the structural similarity index measure SSIM [50] are employed. The proposed method has the highest $Q_{Y}$ value and the second highest according to SSIM, which indicates high fused image quality, regarding structural similarity. DenseFuse [31] has highest SSIM value for the Lytro dataset. The proposed CRF-Guided method has the highest value on the human perception inspired fusion metric CB [49], which implies that perceptually the produced results by the method are the most pleasing to the human eye. According to the blind image quality metric NIQE [51], CRF-Guided has the lowest value and thus the best fused image quality. Lastly, for the blind image quality Entropy, GBM [36] has the highest score and CRF-Guided has the second highest score. Overall for the Lytro dataset [35] of perfectly registered color input images, the proposed CRF-Guided method outperforms the compared state-of-the art image fusion methods in most metrics.

Table 2 includes the quantitative evaluation of the compared methods for the grayscale dataset [3]. The CRF-Guided fusion method outperforms the compared state-of-the-art methods, in terms of metrics MI [45], Qg [47], $Q^{AB/F}$ [46], $Q_{Y}$ [48], CB [49] and SSIM [50] and has the second lowest score for the NIQE [51] metric and the second highest Entropy value. More precisely, since CRF-Guided has the highest Mutual Information [45], it preserves better the original information compared to the other methods. The highest value of CRF-Guided in $Q_{g}$ [47] and $Q^{AB/F}$ [46] indicate that the proposed method preserves better the edges of the input images, compared to the state-of-the-art methods. Moreover, the structural information of the original images is best preserved in the CRF-Guided method, since both $Q_{y}$ [48] and SSIM [50] have the highest value for the proposed method. According to the human perception inspired fusion metric CB [49], CRF-Guided has the best fused image quality. For the NIQE [51] metric, the method dchwt [17] has the lowest score and the proposed method has the second lowest value. The method GBM [36] has the highest entropy value for the grayscale dataset. Overall, the proposed method has the highest fused image compared to the state of the art methods for the grayscale dataset [3].

In summary, according to the 8 metrics used for quantitative evaluation, the proposed CRF-Guided method has the best performance compared to 13 state-of-the art image fusion methods for both public datasets: the Lytro dataset [35] and the grayscale dataset [3].

### 4.2. Qualitative Evaluation

**Figure 11:**
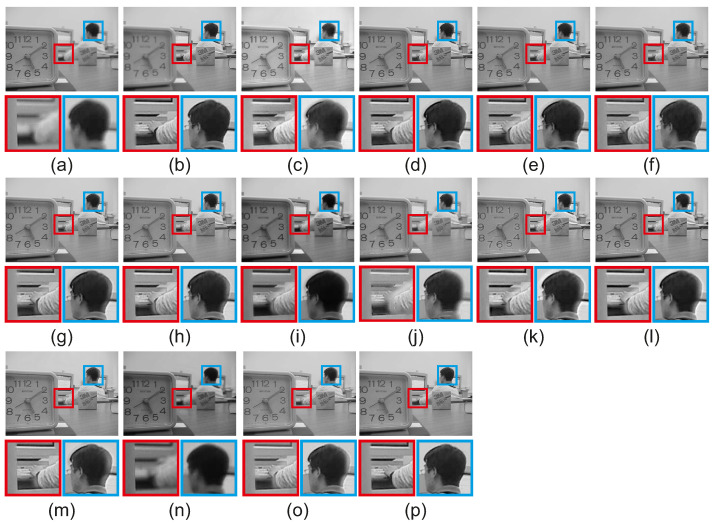
Fused results for the scene ‘Lab’ of the grayscale dataset [3]. (**a**) Source 1, (**b**) Source 2, (**c**) GBM [36], (**d**) NSCT [14], (**e**) ICA [11], (**f**) DCHWT [17], (**g**) ASR [12], (**h**) IFCNN [30], (**i**) DenseFuse [31], (**j**) acof [37], (**k**) CFL [38], (**l**) ConvCFL [39], (**m**) DTNP [40], (**n**) MLCF [41], (**o**) Joint [42], (**p**) CRFGuided.

In this section, we perform a visual comparison between the tested methods. Figure 11 includes the fused results of the compared methods for the scene ‘Lab’ of the grayscale dataset [3]. The compared methods GBM [36], NSCT [14], ICA [11], DCHWT [17], ASR [12], IFCNN [30], DenseFuse [31], acof [37], CFL [38], ConvCFL [39], DTNP [40], MLCF [41] and Joint [42], all feature visible artifacts in the area of the head. Moreover, these methods cannot accurately preserve the boundary of the clock in the red rectangle. MLCF cannot accurately capture the boundaries of the well-focused and out-of-focus pixels. NSCT [14], ICA [11], IFCNN [30], DenseFuse [31], acof [37], CFL [38], ConvCFL [39] also feature artifacts around the arm, included in the red rectangle area. The proposed CRF-Guided method has the highest fused image quality for the area of the head, without introducing artifacts during fusion. Furthermore, the boundary of the clock is best preserved in the CRF-Guided fusion method, compared to the state-of-the-art methods. Moreover, the CRF-Guided fusion method does not introduce artifacts in the area of the red rectangle around the arm. The proposed CRF-Guided method does not have artifacts during fusion and has the highest visual image quality for the ‘Lab’ scene.

**Figure 12:**
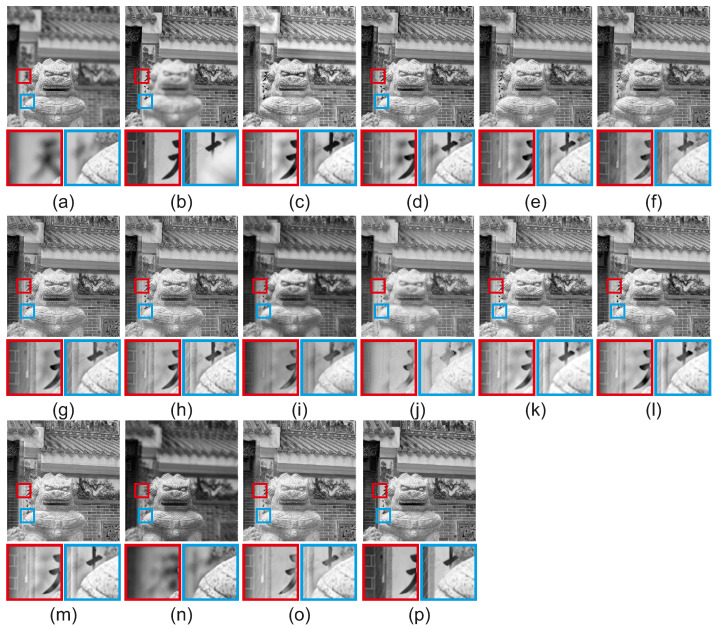
Fused results for the scene ‘Temple’ of the grayscale dataset [3]. (**a**) Source 1, (**b**) Source 2, (**c**) GBM [36], (**d**) NSCT [14], (**e**) ICA [11], (**f**) DCHWT [17], (**g**) ASR [12], (**h**) IFCNN [30], (**i**) DenseFuse [31], (**j**) acof [37], (**k**) CFL [38], (**l**) ConvCFL [39], (**m**) DTNP [40], (**n**) MLCF [41], (**o**) Joint [42], (**p**) CRFGuided.

Figure 12 includes the resulting fused images of the proposed and the compared methods for the scene ‘Temple’ of the grayscale dataset [3]. Two regions are selected for magnification to assess with qualitative evaluation. GBM [36], NSCT [14], ICA [11], DCHWT [17], ASR [12], IFCNN [30], DenseFuse [31], acof [37], CFL [38], ConvCFL [39], DTNP [40], MLCF [41] and Joint [42] all have visible artifacts in both regions of the red and the blue rectangles. Moreover, they cannot accurately preserve the boundary of the well-focused and out-of-focus pixels. The proposed CRF-Guided method preserves accurately the boundaries between the well-focused and out-of-focus pixels for both regions without introducing artifacts, compared to the other multi-focus image fusion methods. CRF-Guided features the best fused image quality for the scene ‘Temple’. Qualitative evaluation indicates that the proposed CRF-Guided method has the best visual fused image quality, without introducing artifacts during fusion, compared to 13 state-of-the art methods.

**Figure 13:**
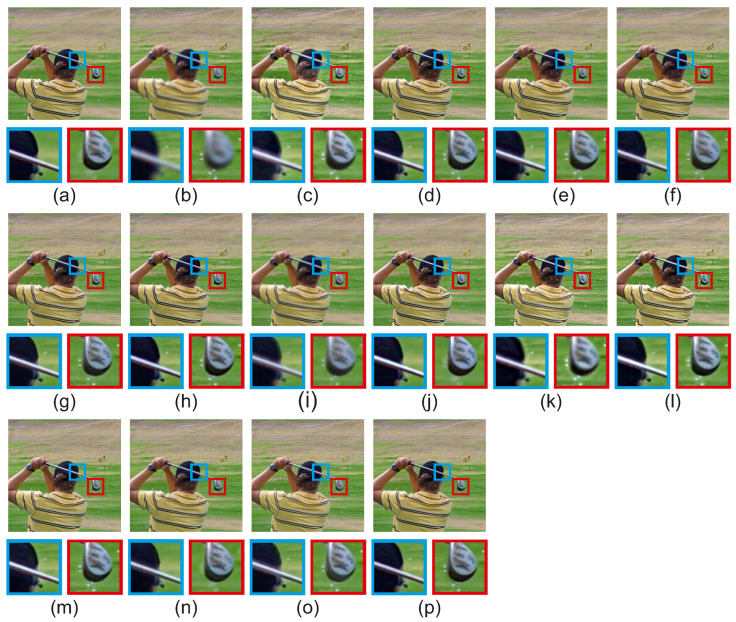
Fused results for the scene ‘Golfer’ of the Lytro dataset [35]. (**a**) Source 1, (**b**) Source 2, (**c**) GBM [36], (**d**) NSCT [14], (**e**) ICA [11], (**f**) DCHWT [17], (**g**) ASR [12], (**h**) IFCNN [30], (**i**) DenseFuse [31], (**j**) acof [37], (**k**) CFL [38], (**l**) ConvCFL [39], (**m**) DTNP [40], (**n**) MLCF [41], (**o**) Joint [42], (**p**) CRFGuided.

Figure 13 includes the qualitative evaluation of the compared methods for the scene ‘Golfer’ of the Lytro dataset [35]. CFL [38] and ConvCFL [39] produce artifacts around the boundary of well-focused and out-of-focus pixels in both regions. The boundary of the well-focused pixels isn’t well preserved in GBM [36], NSCT [14], ICA [11], DCHWT [17], ASR [12], IFCNN [30], DenseFuse [31], acof [37], CFL [38], ConvCFL [39], DTNP [40], MLCF [41] and joint [42], while on the proposed CRFGuided the fused image is better preserved. Methods acof [37], mlcf [41] cannot accurately capture the boundary of well-focused and out-of-focus pixels in both regions. NSCT [14], DenseFuse [31], acof [37], DTNP [40] and MLCF [41] cannot preserve accurately the boundaries between the well-focused and out-of-focus pixels in the area of the red rectangle. The proposed CRFGuided method has the highest visual quality for both regions for the ‘Golfer’ scene of the Lytro [35] dataset, preserving best the boundary of well-focused and out-of-focus pixels, without introducing artifacts during fusion.

**Figure 14:**
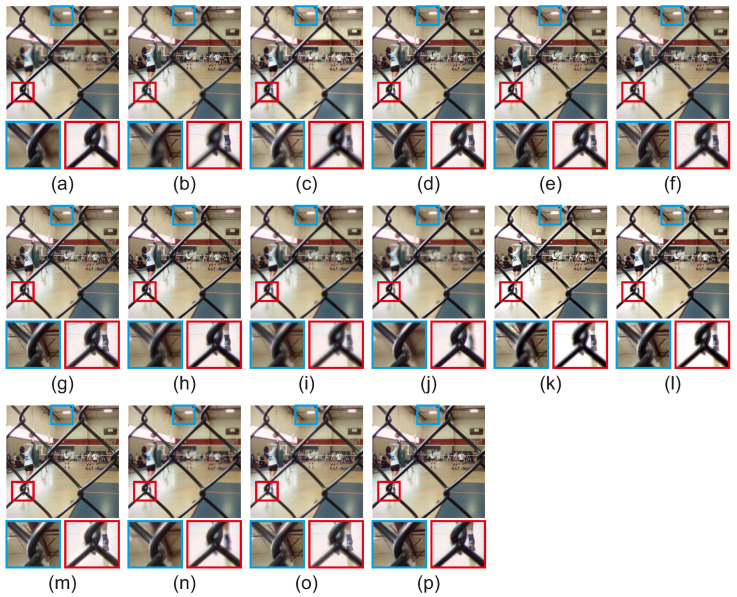
Fused results for the scene ‘Volley’ of the Lytro dataset [35]. (**a**) Source 1, (**b**) Source 2, (**c**) GBM [36], (**d**) NSCT [14], (**e**) ICA [11], (**f**) DCHWT [17], (**g**) ASR [12], (**h**) IFCNN [30], (**i**) DenseFuse [31], (**j**) acof [37], (**k**) CFL [38], (**l**) ConvCFL [39], (**m**) DTNP [40], (**n**) MLCF [41], (**o**) Joint [42], (**p**) CRFGuided.

Figure 14 features the qualitative evaluation for the ‘Volley’ scene of the Lytro [35] dataset. Two regions were selected with magnification. For the blue region, the boundaries of well-focused and out-of-focus pixels in methods GBM [36], NSCT [14], ICA [11], DCHWT [17], IFCNN [30], DenseFuse [31], acof [37], CFL [38], ConvCFL [39], DTNP [40], MLCF [41] are not accurately preserved. Methods acof [37] and MLCF [41] can not preserve accurately the boundaries of well-focused and out-of-focus pixels in both regions. For the red region, Joint [42] produces color degradation and lower contrast. Moreover, GBM [36], NSCT [14], ICA [11], DCHWT [17], ASR [12], IFCNN [30], DenseFuse [31], acof [37], CFL [38], ConvCFL [39], DTNP [40], MLCF [41] can not preserve well the boundary of well-focused and out-of-focus pixels and the back shoe is not well-focused. The proposed CRFGuided preserves best the boundary between well-focused and out-of-focus pixels for both regions for the ‘Volley’ scene of the Lytro dataset [35], resulting in a fused image of high quality without introducing artifacts during fusion.

According to the previous qualitative evaluation, the proposed CRF-Guided fusion method produces fused images of high quality, preserving best the boundary of well-focused and out-of-focus pixels without introducing artifacts during fusion.

### 4.3. Complexity

We analyzed the computational complexity of the proposed and compared image fusion methods. The average execution time on the Lytro dataset of the compared methods are included in Table 3. The included times were computed on an Intel^®^ Core^TM^ i9 2.9 GHz processor with 16 GB RAM and a 64-bit operating system. IFCNN [30] and DenseFuse [31] were executed on an NVIDIA GeForce RTX 2080 with Max-Q Design.

The two deep learning-based approaches IFCNN and DenseFuse have very small execution times, due to their parallel implementation on a GPU. The remaining methods were implemented on MATLAB v2021b. The proposed CRF-Guided was implemented on MATLAB without any code optimization. Nonetheless, its average execution time of 31 s compares favorably with the fastest methods, being the 6th method (excluding the IFCNN and DenseFuse), but with the best overall qualitative performance. Thus, the best qualitative fortunately implies a medium computational complexity.

## 5. Conclusions

A novel transform domain multi-focus image fusion method is introduced in this paper. The proposed CRF-Guided fusion takes advantage of the CRF minimization and the labels are used to guide the fusion of both low frequency and the ICA transform coefficients and thus the high frequency. CRF-Guided fusion supports image denoising during fusion, by applying coefficient shrinkage. Quantitative and qualitative evaluation demonstrate that CRF-Guided fusion outperforms state-of-the-art multi-focus image fusion methods. Limitations of the proposed CRF-Guided fusion method include the selection of the transform domain and the hand-crafted design of the unary and smoothness potential functions for the energy minimization problem. Future work includes the application of CRF-Guided fusion in different transform domains and learning the unary and smoothness potential function with deep learning networks.

## Figures and Tables

**Table 1:** Objective evaluation for the Lytro dataset [35]. Lower values for NIQE indicate better fused image quality, while for rest metrics higher values indicate better fused image quality.

Methods	MI [45]	Qg [47]	$Q^{\mathrm{AB}/F}$ [46]	Qy [48]	CB [49]	SSIM [50]	NIQE [51]	Entropy
ASR [12]	7.1310	0.7510	0.7013	0.9691	0.7264	0.8437	3.4591	7.5217
NSCT [14]	7.1986	0.7502	0.6960	0.9649	0.7527	0.8432	3.4479	7.5309
GBM [36]	3.8813	0.7172	0.6202	0.8554	0.6159	0.7932	3.0434	7.5684
ICA [11]	6.8769	0.7393	0.6741	0.9512	0.7088	0.8534	3.3915	7.5267
IFCNN [30]	7.0400	0.7337	0.6628	0.9522	0.7292	0.8440	3.4623	7.5319
DenseFuse [31]	6.2048	0.5532	0.4694	0.8141	0.6037	**0.8651**	3.3953	7.4681
dchwt [17]	6.7298	0.7184	0.6078	0.9202	0.6924	0.8526	3.2976	7.5205
acof [37]	7.2675	0.5287	0.5112	0.9475	0.6387	0.8260	4.6501	7.4901
cfl [38]	5.6254	0.6576	0.5746	0.8827	0.6323	0.8158	3.4033	**7.5734**
ConvCFL [39]	5.9742	0.6916	0.5864	0.8869	0.6643	0.8396	3.7099	7.5581
DTNP [40]	6.7854	0.7431	0.6779	0.9566	0.7347	0.8390	3.4198	7.5298
mlcf [41]	6.4414	0.5377	0.5147	0.8593	0.6259	0.8564	3.8699	7.4906
joint [42]	6.9991	0.7435	0.6970	0.9621	0.7176	0.8426	3.3935	7.5200
CRFGuided	**7.3639**	**0.7534**	**0.7143**	**0.9851**	**0.7557**	0.8601	**3.0336**	7.5697

**Table 2:** Objective evaluation for the grayscale dataset [3]. Lower values for NIQE indicate better fused image quality, while for rest metrics higher values indicate better fused image quality.

Methods	MI [45]	Qg [47]	$Q^{\mathrm{AB}/F}$ [46]	Qy [48]	CB [49]	SSIM [50]	NIQE [51]	Entropy
ASR [12]	6.3790	0.7192	0.6721	0.9541	0.7057	0.8150	5.5111	7.3262
NSCT [14]	6.2947	0.7074	0.6593	0.9439	0.7284	0.8161	5.3080	7.3451
GBM [36]	3.5292	0.6729	0.5826	0.8275	0.6005	0.7503	5.0053	**7.5298**
ICA [11]	6.0174	0.6945	0.6507	0.9313	0.6996	0.8302	5.2144	7.3449
IFCNN [30]	5.9641	0.6743	0.6074	0.9118	0.6725	0.8230	5.4436	7.3435
DenseFuse [31]	6.0467	0.6139	0.5798	0.8517	0.6275	0.8351	5.2584	7.3739
dchwt [17]	5.9965	0.6781	0.5810	0.8997	0.6752	0.8244	**4.9713**	7.3396
acof [37]	6.5748	0.5594	0.5543	0.8691	0.6183	0.8098	5.1625	7.3088
cfl [38]	4.8158	0.5985	0.5327	0.8548	0.6138	0.7966	5.5156	7.4403
ConvCFL [39]	5.3014	0.6510	0.5619	0.8640	0.6558	0.8234	5.5023	7.3895
DTNP [40]	6.0911	0.6966	0.6357	0.9296	0.7056	0.8119	5.2817	7.3496
mlcf [41]	6.3294	0.5912	0.5890	0.9274	0.6594	0.8040	5.2670	7.3176
joint [42]	6.6541	0.7212	0.6775	0.9553	0.7234	0.8102	5.4543	7.3239
CRFGuided	**6.6740**	**0.7290**	**0.6903**	**0.9798**	**0.7337**	**0.8356**	5.0001	7.3928

**Table 3:** Average running time of compared methods for input image pairs of size $\left[520\times 520\right]$.

Methods	Time (s)
GBM [36]	2.43 s
NSCT [14]	87.27 s
ICA [11]	24.02 s
DCHWT [17]	18.59 s
ASR [12]	1204.92 s
IFCNN [30]	0.22 s
DenseFuse [31]	0.41 s
acof [37]	9.91 s
CFL [38]	23.69 s
ConvCFL [39]	138.42 s
DTNP [40]	420 s
MLCF [41]	53.11 s
Joint [42]	83.09 s
CRF-Guided	31.00 s

## Data Availability

Data sharing is not applicable to this article.

## References

[1] Liu Y., Chen X., Wang Z., Wang Z.J., Ward R.K., Wang X. (2018). Deep learning for pixel-level image fusion: Recent advances and future prospects. Inf. Fusion.

[2] Bai X., Zhang Y., Zhou F., Xue B. (2015). Quadtree-based multi-focus image fusion using a weighted focus-measure. Inf. Fusion.

[3] Zhang Y., Bai X., Wang T. (2017). Boundary finding based multi-focus image fusion through multi-scale morphological focus-measure. Inf. Fusion.

[4] Liu Y., Liu S., Wang Z. (2015). Multi-focus image fusion with dense SIFT. Inf. Fusion.

[5] Qiu X., Li M., Zhang L., Yuan X. (2019). Guided filter-based multi-focus image fusion through focus region detection. Signal Process. Image Commun..

[6] Li M., Cai W., Tan Z. (2006). A region-based multi-sensor image fusion scheme using pulse-coupled neural network. Pattern Recognit. Lett..

[7] Li S., Kang X., Hu J., Yang B. (2013). Image matting for fusion of multi-focus images in dynamic scenes. Inf. Fusion.

[8] Singh S., Singh H., Mittal N., Hussien A.G., Sroubek F. (2022). A feature level image fusion for Night-Vision context enhancement using Arithmetic optimization algorithm based image segmentation. Expert Syst. Appl..

[9] Singh S., Mittal N., Singh H. (2022). A feature level image fusion for IR and visible image using mNMRA based segmentation. Neural Comput. Appl..

[10] Hyvärinen A., Hurri J., Hoyer P.O. (2009). Independent Component Analysis. Natural Image Statistics: A Probabilistic Approach to Early Computational Vision.

[11] Mitianoudis N., Stathaki T. (2007). Pixel-based and region-based image fusion schemes using ICA bases. Inf. Fusion.

[12] Liu Y., Wang Z. (2015). Simultaneous image fusion and denoising with adaptive sparse representation. IET Image Process..

[13] Liu Y., Chen X., Ward R.K., Wang Z.J. (2016). Image Fusion with convolutional sparse representation. IEEE Signal Process. Lett..

[14] Zhang Q., Guo B.l. (2009). Multifocus image fusion using the nonsubsampled contourlet transform. Signal Process..

[15] Liu Y., Liu S., Wang Z. (2015). A general framework for image fusion based on multi-scale transform and sparse representation. Inf. Fusion.

[16] Zhou Z., Li S., Wang B. (2014). Multi-scale weighted gradient-based fusion for multi-focus images. Inf. Fusion.

[17] Shreyamsha Kumar B.K. (2013). Multifocus and multispectral image fusion based on pixel significance using discrete cosine harmonic wavelet transform. Signal Image Video Process..

[18] Qin X., Ban Y., Wu P., Yang B., Liu S., Yin L., Liu M., Zheng W. (2022). Improved Image Fusion Method Based on Sparse Decomposition. Electronics.

[19] Jagtap N.S., Thepade S.D. (2021). High-quality image multi-focus fusion to address ringing and blurring artifacts without loss of information. Vis. Comput..

[20] Singh H., Cristobal G., Bueno G., Blanco S., Singh S., Hrisheekesha P.N., Mittal N. (2022). Multi-exposure microscopic image fusion-based detail enhancement algorithm. Ultramicroscopy.

[21] Bouzos O., Andreadis I., Mitianoudis N. (2019). Conditional random field model for robust multi-focus image fusion. IEEE Trans. Image Process..

[22] Chai Y., Li H., Li Z. (2011). Multifocus image fusion scheme using focused region detection and multiresolution. Opt. Commun..

[23] He K., Zhou D., Zhang X., Nie R. (2018). Multi-focus: Focused region finding and multi-scale transform for image fusion. Neurocomputing.

[24] Singh S., Singh H., Gehlot A., Kaur J., Gagandeep A. (2022). IR and visible image fusion using DWT and bilateral filter. Microsyst. Technol..

[25] Singh S., Mittal N., Singh H. (2020). Multifocus image fusion based on multiresolution pyramid and bilateral filter. IETE J. Res..

[26] Zhang X. (2021). Deep learning-based Multi-focus image fusion: A survey and a comparative study. IEEE Trans. Pattern Anal. Mach. Intell..

[27] Liu Y., Chen X., Peng H., Wang Z. (2017). Multi-focus image fusion with a deep convolutional neural network. Inf. Fusion.

[28] Amin-Naji M., Aghagolzadeh A., Ezoji M. (2019). Ensemble of CNN for multi-focus image fusion. Inf. Fusion.

[29] Tang H., Xiao B., Li W., Wang G. (2018). Pixel convolutional neural network for multi-focus image fusion. Inf. Sci..

[30] Zhang Y., Liu Y., Sun P., Yan H., Zhao X., Zhang L. (2020). IFCNN: A general image fusion framework based on convolutional neural network. Inf. Fusion.

[31] Li H., Wu X.J. (2019). DenseFuse: A fusion approach to infrared and visible images. IEEE Trans. Image Process..

[32] Ma X., Wang Z., Hu S., Kan S. (2022). Multi-focus image fusion based on multi-scale generative adversarial network. Entropy.

[33] Wei B., Feng X., Wang K., Gao B. (2021). The multi-focus-image-fusion method based on convolutional neural network and sparse representation. Entropy.

[34] Boykov Y., Veksler O., Zabih R. (2001). Fast approximate energy minimization via graph cuts. IEEE Trans. Pattern Anal. Mach. Intell..

[35] Nejati M., Samavi S., Shirani S. (2015). Multi-focus image fusion using dictionary-based sparse representation. Inf. Fusion.

[36] Paul S., Sevcenco I.S., Agathoklis P. (2016). Multi-exposure and multi-focus image fusion in gradient domain. J. Circuits Syst. Comput..

[37] Zhu R., Li X., Huang S., Zhang X. (2021). Multimodal medical image fusion using adaptive co-occurrence filter-based decomposition optimization model. Bioinformatics.

[38] Veshki F.G., Ouzir N., Vorobyov S.A., Ollila E. (2022). Multimodal image fusion via coupled feature learning. Signal Process..

[39] Veshki F.G., Vorobyov S.A. Coupled Feature Learning Via Structured Convolutional Sparse Coding for Multimodal Image Fusion. Proceedings of the ICASSP 2022–2022 IEEE International Conference on Acoustics, Speech and Signal Processing (ICASSP).

[40] Li B., Peng H., Wang J. (2021). A novel fusion method based on dynamic threshold neural P systems and nonsubsampled contourlet transform for multi-modality medical images. Signal Process..

[41] Tan W., Thitøn W., Xiang P., Zhou H. (2021). Multi-modal brain image fusion based on multi-level edge-preserving filtering. Biomed. Signal Process. Control..

[42] Li X., Zhou F., Tan H. (2021). Joint image fusion and denoising via three-layer decomposition and sparse representation. Knowl.-Based Syst..

[43] Singh S., Mittal N., Singh H. (2021). Review of various image fusion algorithms and image fusion performance metric. Arch. Comput. Methods Eng..

[44] Singh S., Mittal N., Singh H. (2020). Classification of various image fusion algorithms and their performance evaluation metrics. Computational Intelligence for Machine Learning and Healthcare Informatics.

[45] Hossny M., Nahavandi S., Creighton D. (2008). Comments on ’Information measure for performance of image fusion’. Electron. Lett..

[46] Xydeas C.S., Petrovic V. (2000). Objective image fusion performance measure. Electron. Lett..

[47] Xydeas C.S., Petrovic V.S. (2000). Objective pixel-level image fusion performance measure. Proceedings of the Sensor Fusion: Architectures, Algorithms, and Applications IV.

[48] Yang C., Zhang J.Q., Wang X.R., Liu X. (2008). A novel similarity based quality metric for image fusion. Inf. Fusion.

[49] Chen Y., Blum R.S. (2009). A new automated quality assessment algorithm for image fusion. Image Vis. Comput..

[50] Wang Z., Bovik A., Sheikh H., Simoncelli E. (2004). Image quality assessment: From error visibility to structural similarity. IEEE Trans. Image Process..

[51] Mittal A., Soundararajan R., Bovik A.C. (2013). Making a “Completely Blind” image quality analyzer. IEEE Signal Process. Lett..

